# Childhood emotional abuse and gastrointestinal symptoms among college students: a chain mediation model with physical exercise as the moderating variable and network analysis

**DOI:** 10.3389/fpsyt.2026.1947354

**Published:** 2026-09-23

**Authors:** Menghua Li, Ning Wang, Geng Li, Li Tang, Liying Lan, Ye Wu, Yang Liu

**Affiliations:** 1 Gymnasium of Physical Education Teaching Department, Dalian Minzu University, Dalian, China; 2 Chengdu Vocational University of the Arts, Chengdu, China; 3 School of Psychology, Research Center for Exercise and Brain Science, Shanghai University of Sport, Shanghai, China; 4 Jishou University, Jishou, China; 5 Hospital of Jilin Medical University, Jilin, China; 6 Jilin Medical University, Jilin, China

**Keywords:** childhood emotional abuse, drinking frequency, gastrointestinal symptoms, negative emotional symptoms, network analysis, physical exercise

## Abstract

**Objective:**

To examine the longitudinal association between childhood emotional abuse and gastrointestinal symptoms among college students, assess the serial indirect effects of negative emotional symptoms and drinking frequency, evaluate the moderating role of physical exercise, and use network analysis to identify central symptom nodes and bridge nodes across constructs.

**Methods:**

A three-wave longitudinal study was conducted among 1,896 college students recruited from Hunan, Hubei, Guizhou, and Chongqing, China. Data were collected using measures of childhood trauma, negative emotional symptoms, drinking frequency, physical exercise, and gastrointestinal symptoms. After adjustment for sex, age, and economic status, a moderated serial mediation model was estimated. Network analysis was additionally performed to characterize conditional associations among the study constructs and their individual symptom indicators.

**Results:**

Childhood emotional abuse was significantly and positively associated with subsequent gastrointestinal symptoms. Negative emotional symptoms and drinking frequency each showed a significant specific indirect effect, accounting for 36.23% and 5.10% of the total effect, respectively. The partially time-ordered serial indirect effect along the pathway from childhood emotional abuse to negative emotional symptoms, drinking frequency, and gastrointestinal symptoms accounted for 2.03% of the total effect; none of the bootstrap confidence intervals for the indirect effects included zero. The positive association between childhood emotional abuse and negative emotional symptoms was significantly weaker at higher levels of physical exercise. In the network, anxiety- and stress-related nodes showed relatively high node strength and bridge centrality. A prominent cross-community conditional association was observed between drinking frequency and upper abdominal pain or heartburn, and node-strength estimates showed good stability.

**Conclusion:**

Childhood emotional abuse was closely associated with subsequent gastrointestinal symptoms among college students, and this association may be partly accounted for by negative emotional symptoms and drinking frequency. Physical exercise may buffer the association between early emotional adversity and negative emotional symptoms. Prevention and intervention efforts addressing gastrointestinal health in college students should therefore consider early-trauma screening, emotion regulation, alcohol-risk management, and regular physical exercise.

## 1 Background

Gastrointestinal health problems are common yet often overlooked among college students. Gastrointestinal symptoms (GI), including abdominal pain, reflux, nausea, bloating, and altered bowel habits, may interfere with eating, sleep, academic functioning, and quality of life (1, 2). In the present study, GI refers to self-reported gastrointestinal symptom burden rather than a clinical diagnosis of irritable bowel syndrome (IBS) or another functional gastrointestinal disorder. Accordingly, IBS and functional gastrointestinal disorders are discussed below only when referring to previous studies that specifically examined those conditions. Evidence from Chinese college populations indicates that functional gastrointestinal problems are not uncommon, with prevalence estimates varying across symptom types and student samples (3). A meta-analysis further showed that irritable bowel syndrome affects a notable proportion of Chinese college students and is associated with gender, anxiety, depression, alcohol use, smoking, and sleep problems (4). Longitudinal evidence further suggests a potentially bidirectional association between psychological distress and GI: psychological distress may precede gastrointestinal discomfort, whereas persistent GI may also be accompanied by greater emotional burden (5). Taken together, these findings suggest that GI in college students should be considered within a broader psychological and behavioral context, underscoring the need to identify their potential antecedents and underlying pathways.

In recent years, increasing attention has been directed toward the association between adverse childhood experiences and physical health in early adulthood (6). Childhood emotional abuse (CA) is an important form of childhood trauma and commonly involves persistent denigration, humiliation, rejection, threats, or inadequate emotional care and support. Although CA often leaves no visible injury, its effects may persist through altered self-worth, difficulties in emotion regulation, and heightened stress responsivity (7–10). Stress-sensitization theory proposes that repeated early adversity may increase sensitivity to subsequent stress, thereby intensifying psychological and physiological responses to later challenges (11). Because the college years mark a critical transition from adolescence to early adulthood, vulnerabilities established by early trauma may become especially salient as academic, interpersonal, and environmental demands increase (12, 13). Prior research has linked childhood maltreatment to unexplained GI in adulthood (14). Among different forms of childhood trauma, emotional abuse has shown a particularly strong association with irritable bowel syndrome and greater gastrointestinal symptom burden (15). Clinical research in China has similarly associated CA and emotional neglect with a higher likelihood and greater severity of IBS, as well as poorer quality of life, particularly when adverse experiences persist into adulthood (16). The biopsychosocial model conceptualizes GI as the product of interacting physiological, psychological, and social processes rather than a single organic factor (17). Accordingly, chronic early stress may disrupt stress regulation and gut–brain communication, with downstream effects on gastrointestinal functioning (18, 19). Anxiety, depression, and sustained stress accompanying emotional abuse may also heighten attention to gastrointestinal sensations and promote threatening interpretations of those sensations, thereby increasing subjective symptom burden (20–22). Longitudinal studies likewise suggest a temporal association between childhood emotional trauma and subsequent unexplained gastrointestinal symptoms (23). Overall, existing evidence supports a link between CA and later gastrointestinal health, but multi-wave evidence among college students remains limited. H1: Higher levels of CA will be positively associated with more severe GI among college students.

Negative emotional symptoms (NE) refer to unpleasant affective experiences that arise in response to stress, threat, or frustration, including anxiety, depression, tension, worry, sadness, and persistent psychological stress (24). NE may represent an important psychological pathway through which CA is associated with later GI (25). Gastrointestinal problems and emotional distress frequently co-occur among young people (26). Meta-analytic evidence has likewise linked irritable bowel syndrome with anxiety and depression in both college and clinical populations (27, 28). The process model of emotion regulation suggests that CA may impede the development of effective emotion identification, cognitive reappraisal, and response-modulation strategies. Individuals exposed to CA may consequently rely more on suppression, avoidance, or rumination, thereby sustaining negative affect and physiological arousal that may aggravate gastrointestinal discomfort (29). Empirical findings provide further support for this pathway. Chinese clinical research has associated CA and emotional neglect with a higher likelihood of GI, greater symptom severity, and poorer quality of life, and negative life events in adulthood may further intensify these associations (16). Trauma exposure has also been associated with greater anxiety and depression, with these emotional symptoms showing significant indirect associations with gastrointestinal symptom severity (30). Similarly, anxiety has been found to account for a substantial proportion of the association between adverse childhood experiences and irritable bowel syndrome (31). Longitudinal evidence also indicates that childhood maltreatment may be associated with later GI through neuroticism and subsequent anxiety and depression, while earlier emotional distress is related to abdominal pain and the interference of GI with daily life at follow-up (32). Taken together, NE may constitute an important psychological link between CA and GI. H2: NE will account for a significant indirect association between CA and GI among college students.

Drinking frequency (DF) reflects how often an individual consumes alcohol over a defined period and can help identify recurrent alcohol use among young people who may not meet criteria for alcohol dependence or heavy drinking (33, 34). Previous research suggests that the consequences of CA extend beyond social adjustment and interpersonal functioning to health-related behaviors in early adulthood (35). Persistent denigration, rejection, threats, or inadequate emotional support may weaken behavioral self-regulation and increase reliance on alcohol as a means of avoiding or managing distress (36, 37). During college, wider social networks, stronger peer norms, and greater access to alcohol may further facilitate the translation of early relational adversity into drinking behavior (38). Repeated alcohol use may then become an established coping-related behavioral pattern among students with a history of emotional abuse (39, 40). DF may also be relevant to gastrointestinal health. Alcohol consumption can affect gastrointestinal motility, mucosal integrity, and related digestive functions and has been associated with abdominal discomfort, reflux, nausea, and altered bowel habits (41, 42). Higher-volume or binge drinking has been associated with worse stomach pain, dyspepsia, nausea, and diarrhea, while greater DF and alcohol intake have also been linked to a higher risk of gastroesophageal reflux (43). Thus, more frequent alcohol use may constitute a behavioral pathway linking early emotional adversity with subsequent gastrointestinal discomfort. H3: DF will account for a significant indirect association between CA and GI among college students.

The association between CA and GI may develop through linked psychological and behavioral processes rather than a single mechanism (44–46). The developmental cascade model proposes that adaptation difficulties emerging early in life can spread from one functional domain to another over time, producing linked associations across psychological, behavioral, and physical health domains (47, 48). Repeated childhood experiences of denigration, rejection, or inadequate emotional support may be associated with persistent anxiety, depression, and stress responses (49, 50). NE have shown a significant statistical indirect effect in the association between trauma exposure and GI, suggesting that emotional distress may represent an important psychological link between early trauma and somatic discomfort (51). These emotional difficulties may, in turn, shape subsequent coping behaviors (52). Research has shown that poorer behavioral control may help explain the association between CA and alcohol use in young adulthood (53), while adverse childhood experiences have also been sequentially associated with drinking behaviors through depressive symptoms and coping-related drinking motives (54, 55). Persistent anxiety, depression, or stress may therefore increase the tendency to use alcohol for emotional relief, whereas more frequent alcohol use may subsequently contribute to greater gastrointestinal symptom burden (56, 57). Prospective evidence further suggests that heavier drinking may be followed by worse gastrointestinal symptoms, including diarrhea, nausea, stomach pain, and dyspepsia (58). Accordingly, CA may first be associated with elevated NE, which may then increase DF and, in turn, be associated with more severe GI (49, 50). This sequence is consistent with the cross-domain process proposed by the developmental cascade model, linking early adversity, emotional maladaptation, risk behavior, and physical health. H4: NE and DF will form a significant serial indirect pathway between CA and GI among college students.

Physical exercise (PE) is a modifiable health behavior that may attenuate the association between CA and NE in early adulthood (59). According to the stress-buffering model, protective resources can improve how individuals appraise and cope with stressful events, thereby reducing emotional responses associated with adverse experiences (60). Prolonged exposure to denigration, rejection, threats, or inadequate emotional support during childhood may undermine a sense of security and self-worth and heighten sensitivity to subsequent stress (61, 62). Because academic, interpersonal, and environmental changes cluster during the college years, vulnerabilities established by early emotional trauma may become particularly salient (63). Regular PE may provide a stable behavioral resource for managing these vulnerabilities by facilitating emotion regulation and stress relief (64). It may also strengthen perceived control, self-efficacy, and psychological resilience while reducing rumination and negative self-focused attention (65, 66). Thus, even among students with comparable histories of childhood emotional abuse, those who engage in more PE may be better able to regulate trauma-related anxiety, depression, and stress responses (67). Conversely, students with insufficient PE may have fewer opportunities for emotional release and behavioral activation and may be more prone to avoidance, rumination, and persistent tension, resulting in more severe NE (68). At the physiological level, PE may also support autonomic and stress-response regulation and facilitate post-stress recovery (69, 70). In addition, the regularity and social engagement associated with PE may provide positive experiences and social support that reduce excessive attention to negative affect (71). Taken together, these mechanisms suggest that PE may function as a protective resource that weakens the association between CA and NE. H5: PE will moderate the association between CA and NE among college students, such that the positive association will be weaker at higher levels of PE.

Although previous studies have separately examined associations among childhood emotional abuse, NE, alcohol use, PE, and GI, two important gaps remain. First, most research has focused on single pathways or cross-sectional associations, with few longitudinal studies simultaneously evaluating the serial indirect associations among childhood emotional abuse, NE, drinking frequency, and GI. Second, conventional variable-centered models primarily test statistical paths between aggregate scores and therefore offer limited insight into how specific symptoms connect across distinct systems. Network analysis can identify central and bridge nodes among items measuring GI, NE, and childhood emotional abuse, providing finer-grained evidence on the structure linking psychological, behavioral, and somatic symptoms. To address these limitations, the present three-wave longitudinal study combined a moderated serial mediation model with network analysis to examine both variable-level pathways and symptom-level connections linking CA with subsequent GI. This integrated approach was intended to provide a more concise yet comprehensive account of the psychological and behavioral processes associated with gastrointestinal health among college students. The hypothesized relationships among the study variables are illustrated in **Figure 1**.

**Figure 1:**
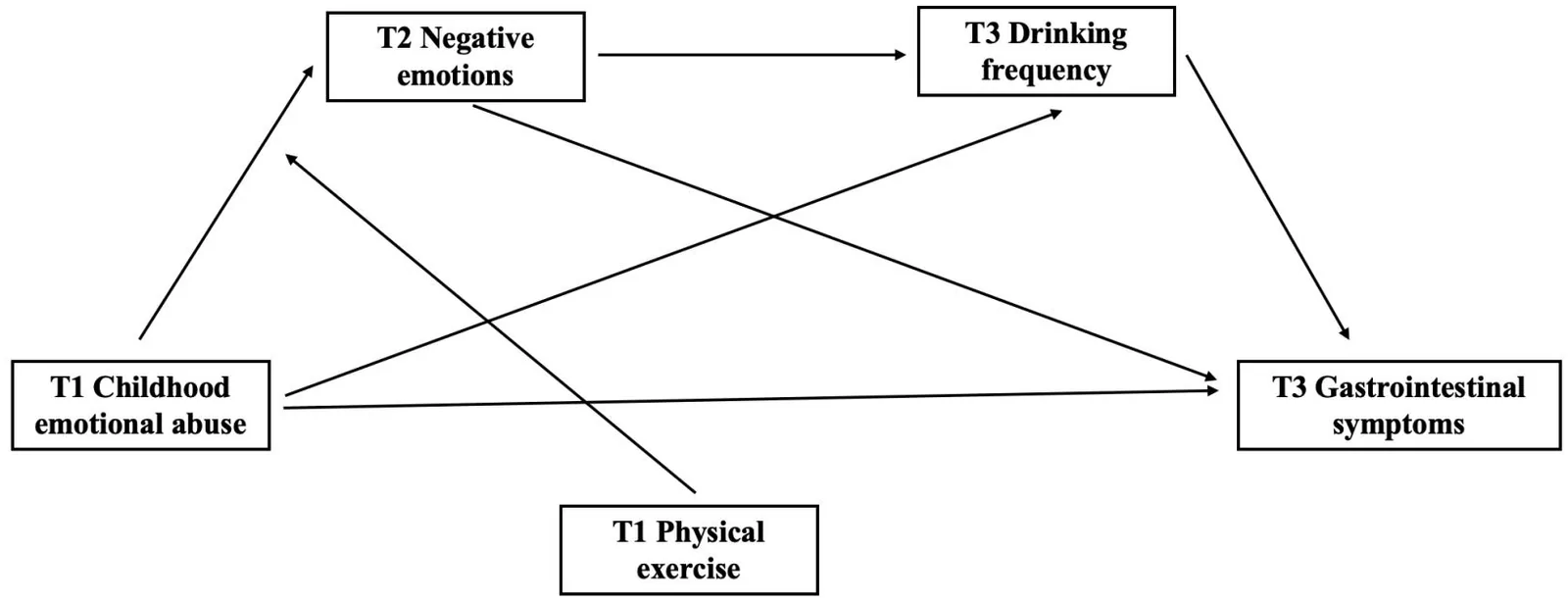
Conceptual moderated serial mediation model.

## 2 Methods

### 2.1 Participants and procedure

This three-wave longitudinal study used convenience sampling to recruit college students from institutions in Hunan, Hubei, and Guizhou provinces and Chongqing Municipality, China. Surveys were administered at the class level, with trained researchers or course instructors coordinating completion of the electronic questionnaires. Before each survey, participants were informed of the study purpose, completion requirements, voluntary nature of participation, and data confidentiality procedures. All participants provided informed consent before completing the questionnaires. Data were collected on June 8, June 15, and July 15, 2026. At Wave 1 (T1), 2,100 responses were collected, primarily assessing CA and PE. At Wave 2 (T2), 2,000 responses were collected, primarily assessing NE. At Wave 3 (T3), 1,900 responses were collected, primarily assessing DF and GI. Unique study codes were used to match responses across the three waves. After records with missing study variables, unmatched data, clearly patterned responses, or logical inconsistencies were excluded, 1,896 participants were retained in the final analytic sample, corresponding to 90.29% of the original T1 sample. Attrition was 4.76% from T1 to T2 and 5.00% from T2 to T3; an additional 0.21% of T3 questionnaires were excluded during data cleaning. Participants had a mean age of 19.24 years (SD = 1.26); 868 were male (45.78%) and 1,028 were female (54.22%). Participant characteristics are summarized in **Table 1**. Eligibility criteria were enrollment as a full-time undergraduate, sufficient reading comprehension to complete the questionnaire independently, voluntary participation with informed consent, and successful matching of responses across all three waves using the study code. Exclusion criteria were ineligibility for the target population, failure to complete any survey wave, unsuccessful matching across waves, missing data for any study variable, or evidence of patterned, careless, logically inconsistent, or implausibly rapid responding.

**Table 1:** Participant characteristics (n = 1,896).

Characteristic	Category	n	Percentage (%)
Gender	Male	868	45.78
	Female	1028	54.22
Residence	Rural	990	52.22
	Urban	906	47.78
Only-child status	Yes	483	25.47
	No	1413	74.53
Left-behind experience	Yes	1033	54.48
	No	863	45.52
Maternal education	Primary school or below	232	12.24
	Junior high school	678	35.76
	Senior high school	504	26.58
	University	296	15.61
	Postgraduate	34	1.79
	Unknown	152	8.02
Paternal education	Primary school or below	343	18.09
	Junior high school	687	36.23
	Senior high school	439	23.15
	University	238	12.55
	Postgraduate	33	1.74
	Unknown	156	8.24

### 2.2 Measures

#### 2.2.1 Childhood emotional abuse

Childhood emotional abuse was assessed using four items selected from the emotional abuse and emotional neglect subscales of the Chinese revision of the Childhood Trauma Questionnaire-Short Form (CTQ-SF) Zhao et al. (72). Items were rated on a 5-point Likert scale ranging from 1 (never) to 5 (always). Items drawn from the childhood emotional neglect subscale were reverse-scored before the four item scores were summed, yielding a study-specific Childhood emotional abuse composite ranging from 4 to 20; higher scores indicated higher levels on this composite. In the present sample, Cronbach’s alpha was 0.849 and the Kaiser-Meyer-Olkin (KMO) value was 0.770, supporting adequate internal consistency and sampling adequacy (73, 74).

#### 2.2.2 Gastrointestinal symptoms

Gastrointestinal symptom severity was assessed using an adapted eight-item measure based on the Gastrointestinal Symptom Rating Scale (GSRS) Svedlund et al. (75). The GSRS was originally developed by Svedlund and colleagues as a disease-specific patient-reported outcome measure of gastrointestinal symptoms and their severity, and its psychometric properties have subsequently been examined in other populations. Chinese-language versions have also demonstrated acceptable reliability and validity in Chinese clinical samples. The 8-item checklist used in this study assessed upper abdominal pain or heartburn, acid regurgitation or nausea, abdominal rumbling or bloating, belching or excessive flatulence, constipation or diarrhea, hard or loose stools, urgency to defecate, and a sensation of incomplete evacuation after defecation. Each item was rated on a 7-point Likert scale from 1 (not at all) to 7 (very severe). Item scores were summed to produce a total score of 8-56, with higher scores indicating more severe gastrointestinal symptoms. In the present sample, Cronbach’s alpha was 0.928 and the KMO value was 0.919. Confirmatory factor analysis produced χ²/df = 9.916, CFI = 0.988, TLI = 0.977, RMSEA = 0.069, and SRMR = 0.022. The CFI, TLI, RMSEA, and SRMR were broadly consistent with acceptable-to-good fit, although the χ²/df ratio was elevated.

#### 2.2.3 Negative emotional symptoms

Negative emotional symptoms were assessed using a six-item short form derived from the Depression Anxiety Stress Scales (DASS). The original instrument was developed by Lovibond and Lovibond (76) and was subsequently translated and adapted for Chinese college students Gong et al. (77). Zhang et al. (78) later used network and factor analyses to select six items with high expected influence and factor loadings, yielding a briefer DASS-6 form. For scoring in the present study, two items were treated as representing each of the three domains of depression, anxiety, and stress. Items were rated on a 4-point Likert scale from 0 (not applicable) to 3 (always applicable). The six item scores were summed to obtain a negative emotional symptom score ranging from 0 to 18, with higher scores indicating more severe negative emotional symptoms. In the present sample, Cronbach’s alpha was 0.927 and the KMO value was 0.918, indicating strong internal consistency and sampling adequacy for factor analysis.

#### 2.2.4 Drinking frequency

DF during the previous month was assessed with a single item adapted from the drinking-frequency question in the Alcohol Use Disorders Identification Test-Consumption (AUDIT-C) (79). The AUDIT-C is the brief consumption component of the AUDIT and is used to screen for hazardous alcohol use and alcohol-related problems; its drinking-frequency item has also been widely used to characterize alcohol use. Participants were asked, “During the past month, how often did you drink alcohol?” Response options were scored as 1 (not at all), 2 (once), 3 (2–4 times), 4 (2–3 times per week), and 5 (4 or more times per week). Higher scores indicated more frequent drinking during the previous month.

#### 2.2.5 Physical exercise

Physical exercise was assessed with a single item asking, “During the past 7 days, on how many days did you exercise or engage in at least 30 minutes of physical activity that made you sweat or breathe hard?” Responses ranged from 0 to 7 days (80), with higher scores indicating more frequent physical exercise. This single-item measure was brief and imposed minimal response burden, making it suitable for a large-sample survey. Previous research suggests that comparable single-item measures have acceptable test-retest reliability and criterion-related validity for assessing physical activity at a single time point, and that their responsiveness to change in adult samples may approximate that of accelerometer-based measures (81, 82).

### 2.3 Statistical analysis

Means, standard deviations, and Pearson correlation coefficients were calculated first using the variables in their original scoring metrics. PROCESS Model 6 was then specified to examine the serial indirect associations linking T1 CA to T3 GI through T2 NE and T3 DF. PROCESS Model 83 was used to examine whether T1 PE moderated the path from T1 CA to T2 NE within the conditional process model. Gender, age, and economic status were included as covariates. For the PROCESS analyses, the focal continuous study variables were z-standardized before model estimation, whereas the covariates retained their original coding. In Model 83, PROCESS additionally mean-centered the standardized T1 CA and T1 PE scores before constructing the CA × PE interaction term. Because z-standardization had already centered these variables at approximately zero, this additional centering did not materially alter their scale and was used only in constructing and interpreting the interaction term. Indirect effects, conditional indirect effects, and the index of moderated mediation were estimated using 5,000 bootstrap resamples. An effect was considered statistically distinguishable from zero when its 95% confidence interval did not include zero. Simple slopes were evaluated at low (M - 1 SD), mean (M), and high (M + 1 SD) levels of PE. All tests were two-sided, with statistical significance set at p < 0.05. Network analyses were conducted in R version 4.5.2. A regularized partial-correlation network was estimated using EBICglasso. The correlation matrix was constructed using qgraph::cor_auto to accommodate continuous and ordinal variables, and the EBIC tuning parameter was set to γ = 0.50. Node centrality was evaluated using strength, whereas cross-community connectivity was assessed using bridge strength and one-step bridge expected influence. Nonparametric bootstrap procedures were used to examine the accuracy of edge-weight estimates, and case-dropping bootstrap procedures were used to assess the stability of node strength.

## 3 Results

### 3.1 Assessment of common method bias

Common method bias was assessed using Harman’s single-factor test. An unrotated principal component analysis extracted four factors with eigenvalues greater than 1, and the first factor accounted for 38.99% of the total variance, below the conventional 40% threshold. A confirmatory single-factor model also showed poor fit (χ²/df = 63.56, CFI = 0.556, TLI = 0.503, RMSEA = 0.182, and SRMR = 0.150), indicating that a single latent factor did not adequately account for all measured indicators. These findings did not indicate a dominant single-factor explanation, although they cannot rule out common method bias.

### 3.2 Descriptive statistics and correlations

As shown in **Table 2**, T1 CA was positively correlated with T2 NE (r = 0.365, p < 0.01), T3 DF (r = 0.147, p < 0.01), and T3 GI (r = 0.342, p < 0.01). T2 NE were positively correlated with both T3 DF (r = 0.153, p < 0.01) and T3 GI (r = 0.438, p < 0.01). T3 DF was also positively correlated with T3 GI (r = 0.196, p < 0.01). T1 PE was negatively correlated with T2 NE (r = −0.068, p < 0.01) and T3 GI (r = −0.058, p < 0.05).

**Table 2:** Descriptive statistics and correlations among study variables.

Variable	1	2	3	4	5
1. T1 CA	1				
2. T2 NE	0.365**	1			
3. T3 DF	0.147**	0.153**	1		
4. T1 PE	-0.004	-0.068**	0.014	1	
5. T3 GI	0.342**	0.438**	0.196**	-0.058*	1
M ± SD	8.17 ± 3.12	10.46 ± 3.46	1.53 ± 0.84	3.09 ± 2.11	18.15 ± 9.18

CA, Childhood emotional abuse; NE, Negative emotional symptoms; DF, Drinking frequency; PE, Physical exercise; GI, Gastrointestinal symptoms. *p < 0.05, **p < 0.01.

### 3.3 Serial mediation analysis

After adjustment for gender, age, and economic status, T1 CA was positively associated with T2 NE and T3 DF, while both T2 NE and T3 DF were positively associated with T3 GI (full regression coefficients are provided in **Table 3**). Bootstrap estimates showed that the total effect of T1 CA on T3 GI was 0.340, of which 43.33% was statistically accounted for by the combined indirect effects. The largest specific indirect effect occurred through T2 NE (effect = 0.123, 95% CI [0.098, 0.149]), accounting for 36.23% of the total effect. The indirect effect through T3 DF was smaller (effect = 0.017, 95% CI [0.009, 0.028]; 5.10%), and the partially time-ordered serial indirect association through T2 NE and T3 DF was modest (effect = 0.007, 95% CI [0.003, 0.012]; 2.03%). All bootstrap confidence intervals excluded zero (**Tables 3**, **4**).

**Table 3:** Regression models underlying the serial indirect-effect model involving T2 NE and T3 DF.

Outcome	Predictor	R	R²	F	β	SE	Lower 95% CI	Upper 95% CI
T2 NE	T1 CA	0.368	0.136	74.097***	0.363***	0.022	0.321	0.405
	Gender				0.066	0.044	-0.020	0.153
	Age				0.022	0.017	-0.012	0.056
	Economic status				-0.016	0.013	-0.042	0.009
T3 DF	T1 CA	0.244	0.060	23.936***	0.123***	0.024	0.076	0.170
	T2 NE				0.134***	0.024	0.087	0.181
	Gender				-0.204***	0.046	-0.294	-0.114
	Age				0.015	0.018	-0.021	0.051
	Economic status				0.052***	0.014	0.025	0.079
T3 GI	T1 CA	0.504	0.254	107.312***	0.192***	0.022	0.150	0.235
	T2 NE				0.339***	0.022	0.297	0.381
	T3 DF				0.141***	0.021	0.101	0.181
	Gender				0.177***	0.041	0.096	0.257
	Age				0.019	0.016	-0.013	0.051
	Economic status				-0.022	0.012	-0.046	0.003

CA, Childhood emotional abuse; NE, Negative emotional symptoms; DF, Drinking frequency; PE, Physical exercise; GI, Gastrointestinal symptoms. ***p < 0.001.

**Table 4:** Total, direct, and indirect effects in the serial indirect-effect model.

Effect	Effect (95% CI)	Bootstrap SE	Proportion of total effect (%)
Total effect	0.340 [0.297, 0.382]	0.022	100.00
Direct effect	0.192 [0.150, 0.235]	0.022	56.67
Total indirect effect	0.147 [0.119, 0.179]	0.015	43.33
T1 CA → T2 NE → T3 GI	0.123 [0.098, 0.149]	0.013	36.23
T1 CA → T3 DF → T3 GI	0.017 [0.009, 0.028]	0.005	5.10
T1 CA → T2 NE → T3 DF → T3 GI	0.007 [0.003, 0.012]	0.002	2.03

CA, Childhood emotional abuse; NE, Negative emotional symptoms; DF, Drinking frequency; PE, Physical exercise; GI, Gastrointestinal symptoms; CI, Confidence interval; Bootstrap SE, Bootstrap standard error.

### 3.4 Moderated serial mediation analysis

T1 PE significantly moderated the association between T1 CA and T2 NE (β = -0.064, 95% CI [-0.104, -0.023]), after adjustment for gender, age, and economic status (full model estimates are presented in **Table 5**). Simple-slope analyses showed that the positive association between CA and NE weakened progressively from low PE (effect = 0.427, 95% CI [0.368, 0.487]) to mean PE (effect = 0.364, 95% CI [0.322, 0.406]) and high PE (effect = 0.300, 95% CI [0.243, 0.358]). Thus, PE attenuated, but did not eliminate, the positive association between CA and subsequent NE.

**Table 5:** Regression models for the moderated serial mediation analysis.

Outcome	Predictor	R	R²	F	β	SE	Lower 95% CI	Upper 95% CI
T2 NE	T1 CA (A)	0.379	0.144	52.824***	0.364***	0.021	0.322	0.406
	T1 PE (B)				-0.066**	0.022	-0.109	-0.024
	A × B				-0.064**	0.021	-0.104	-0.023
	Gender				0.032	0.045	-0.056	0.120
	Age				0.023	0.017	-0.011	0.057
	Economic status				-0.014	0.013	-0.040	0.012
T3 DF	T1 CA	0.244	0.060	23.936***	0.123***	0.024	0.076	0.170
	T2 NE				0.134***	0.024	0.087	0.181
	Gender				-0.204***	0.046	-0.294	-0.114
	Age				0.015	0.018	-0.021	0.051
	Economic status				0.052***	0.014	0.025	0.079
T3 GI	T1 CA	0.504	0.254	107.312***	0.192***	0.022	0.150	0.235
	T2 NE				0.339***	0.022	0.297	0.381
	T3 DF				0.141***	0.021	0.101	0.181
	Gender				0.177***	0.041	0.096	0.257
	Age				0.019	0.016	-0.013	0.051
	Economic status				-0.022	0.012	-0.046	0.003

CA, Childhood emotional abuse; NE, Negative emotional symptoms; DF, Drinking frequency; PE, Physical exercise; GI, Gastrointestinal symptoms. **p < 0.01, ***p < 0.001.

Simple-slope analyses were conducted to characterize the moderating role of PE (**Tables 6**, **7**, **Figures 2**, **3**). T1 CA was positively associated with T2 NE at low, mean, and high levels of T1 PE. The association was strongest at a low level of PE, weaker at the mean level, and weakest at a high level. Thus, although the conditional association remained significant across all three levels, its magnitude decreased progressively as PE increased.

**Table 6:** Conditional indirect effects and indices of moderated mediation.

Indirect pathway	T1 PE level	Effect/Index	BootSE	Boot LLCI	Boot ULCI
T1 CA → T2 NE → T3 GI	Low (-1 SD)	0.1449	0.0168	0.1133	0.1784
	Mean	0.1233	0.0133	0.0986	0.1507
	High (+1 SD)	0.1018	0.0160	0.0720	0.1342
	Index of moderated mediation	-0.0215	0.0096	-0.0400	-0.0027
T1 CA → T2 NE → T3 DF → T3 GI	Low (-1 SD)	0.0081	0.0026	0.0036	0.0138
	Mean	0.0069	0.0021	0.0032	0.0115
	High (+1 SD)	0.0057	0.0018	0.0026	0.0096
	Index of moderated mediation	-0.0012	0.0007	-0.0028	-0.0001

CA, Childhood emotional abuse; NE, Negative emotional symptoms; DF, Drinking frequency; PE, Physical exercise; GI, Gastrointestinal symptoms; BootSE, Bootstrap standard error; BootLLCI, Lower limit of the 95% bootstrap confidence interval; BootULCI, Upper limit of the 95% bootstrap confidence interval. Low, mean, and high PE correspond to M − 1 SD, M, and M + 1 SD, respectively. Bootstrap estimates were based on 5,000 resamples.

**Table 7:** Conditional effect of T1 CA on T2 NE at different levels of T1 PE.

T1 PE level	Conditional effect	SE	Lower 95% CI	Upper 95% CI
Low	0.427	0.030	0.368	0.487
Mean	0.364	0.021	0.322	0.406
High	0.300	0.030	0.243	0.358

**Figure 2:**
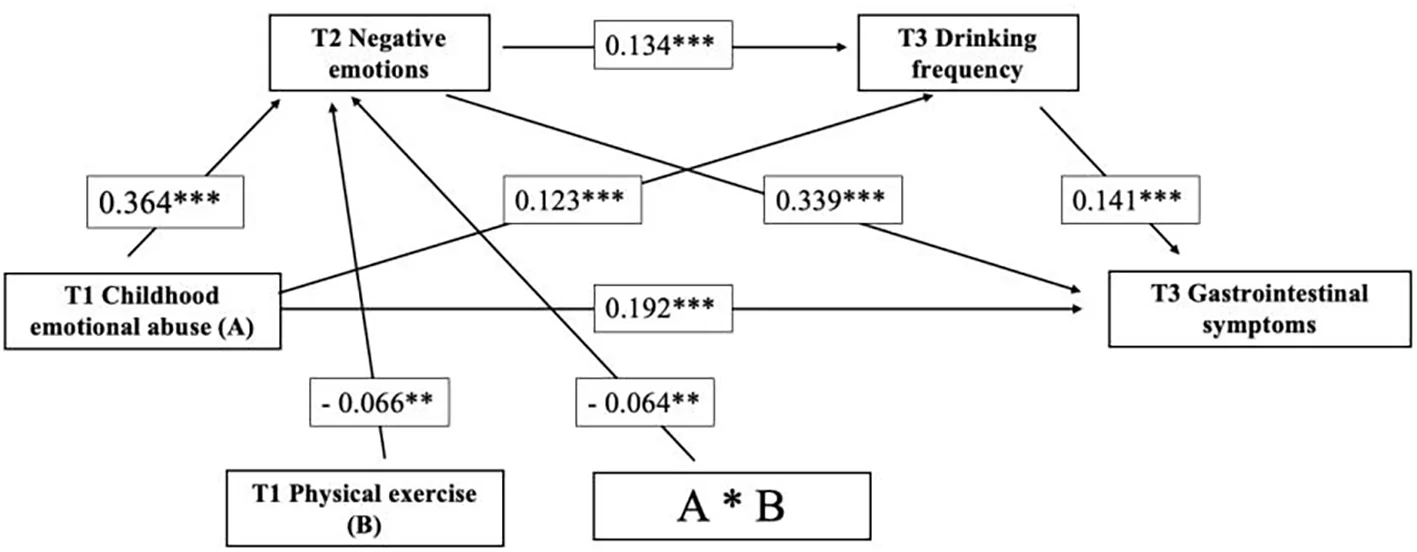
Moderated serial mediation model (**p < 0.01, ***p < 0.001). A * B represents the interaction between T1 childhood emotional abuse (A) and T1 physical exercise (B); **p < 0.01, *p < 0.001.

**Figure 3:**
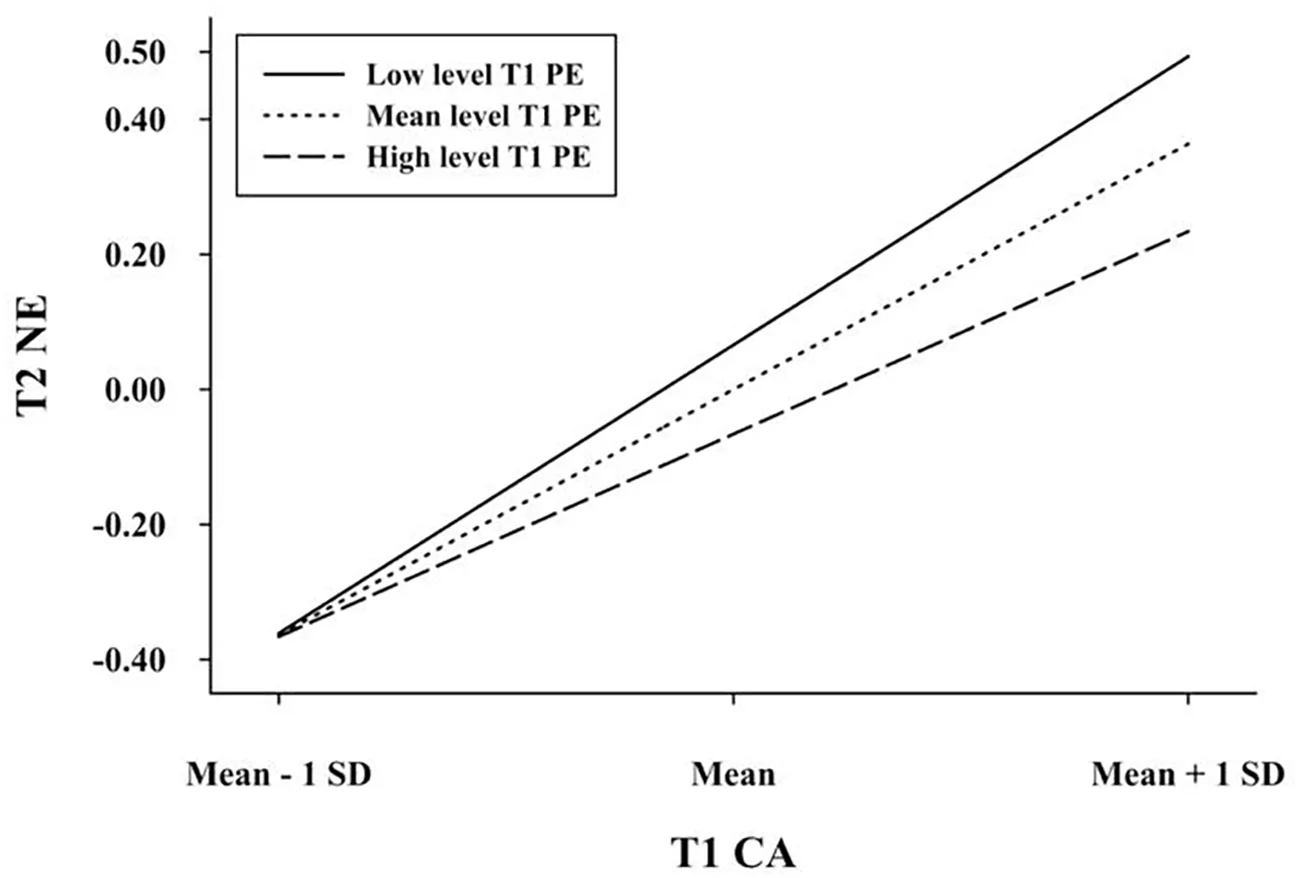
Simple-slope plot for the moderating effect of PE.

### 3.5 Network analysis of study variables

The estimated network displayed a clear community structure (**Figures 4a, b**), with nodes representing T1 childhood emotional abuse, T1 PE, T2 NE, T3 drinking frequency, and T3 GI clustering largely within their respective domains. Gastrointestinal symptom nodes were densely interconnected; particularly strong positive edges were observed among T3 GI2, T3 GI3, and T3 GI4 and among T3 GI5, T3 GI6, T3 GI7, and T3 GI8, indicating substantial conditional associations within the gastrointestinal symptom community. Negative emotional symptom nodes also formed a distinct cluster, with prominent connections among anxiety- and stress-related items. The bridge network identified T3 DF, T3 GI1, T1 CA2, and several T2 negative emotional symptom nodes as cross-community connectors. The edge between T3 DF and T3 GI1 was particularly pronounced. In addition, T2 NE3, T2 NE5, and T2 NE6 showed cross-community connections involving the childhood emotional abuse, drinking frequency, and gastrointestinal symptom communities, indicating that anxiety- and stress-related symptoms occupied bridge positions between these domains.

**Figure 4:**
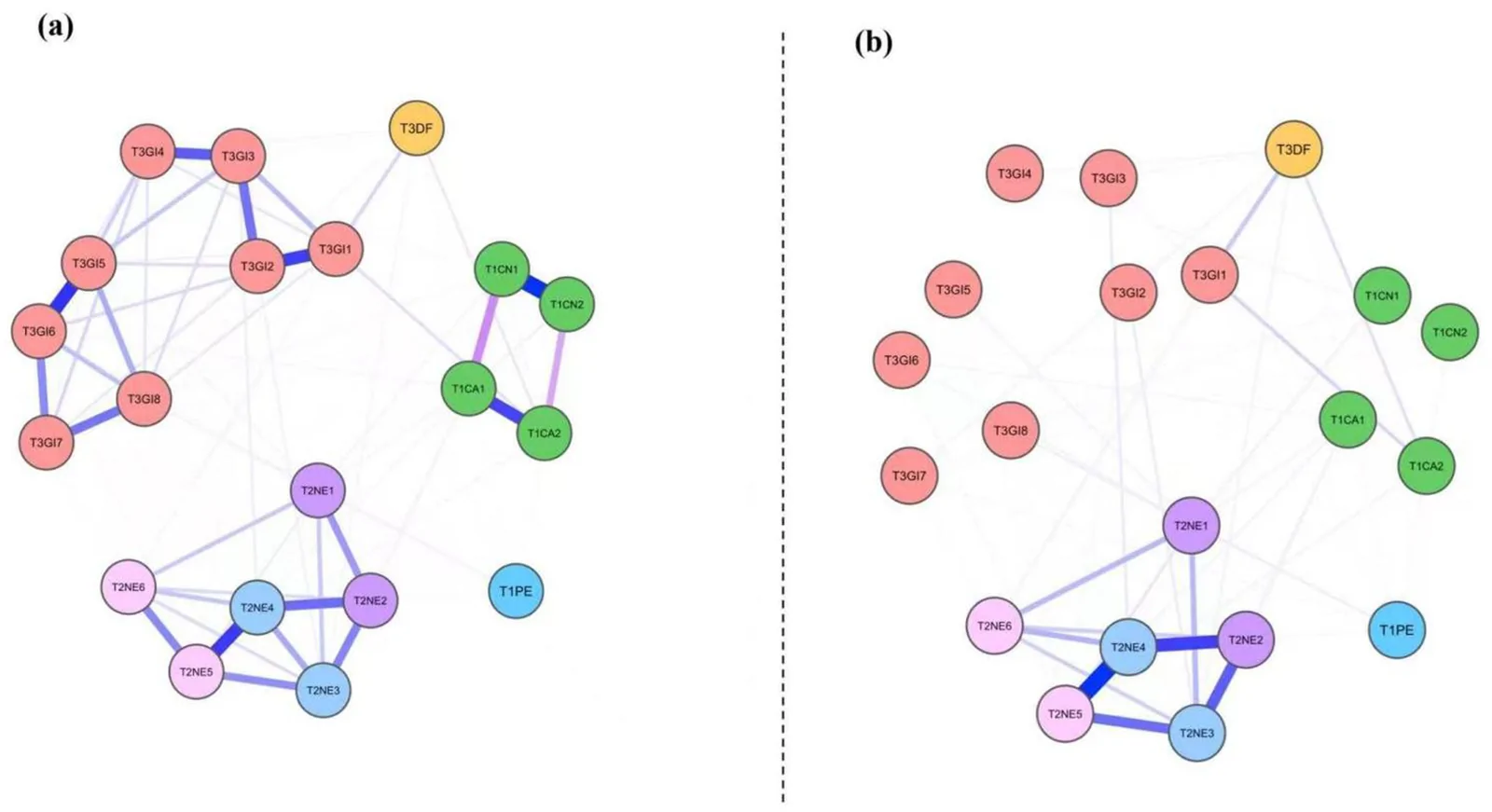
Network structure of the study variables. **(a)** presents the full network, and **(b)** presents the bridge network across variable communities. Node colors denote variable domains: green, T1 childhood emotional abuse; light blue, T1 PE; purple, T2 depression; blue, T2 anxiety; pink, T2 stress; yellow, T3 drinking frequency; and red, T3 GI. Each edge represents the conditional association between two nodes after adjustment for all other nodes in the network; thicker edges indicate stronger associations. Blue edges indicate positive associations, and purple edges indicate negative associations. CA, Childhood emotional abuse; NE, Negative emotional symptoms; DF, Drinking frequency; PE, Physical exercise; GI, Gastrointestinal symptoms.

### 3.6 Node and bridge centrality

Centrality estimates varied markedly across nodes in the full network (**Figures 5a–c**). T2 NE4 had the highest node strength, followed by relatively high values for T3 GI2, T3 GI6, T2 NE5, T3 GI5, T3 GI1, and T2 NE3, indicating that anxiety, stress, and several GI were strongly connected to other nodes. By contrast, T3 DF and T1 PE had comparatively low node strength. T2 NE4 also had the highest bridge strength, followed by T2 NE3, T2 NE5, T2 NE2, T2 NE6, and T2 NE1. Thus, NE —particularly anxiety- and stress-related items—showed the most pronounced connections across variable communities. T3 DF, T3 GI1, T1 CA2, and T1 CA1 also displayed non-negligible bridge strength. The ordering of one-step bridge expected influence was broadly consistent with that of bridge strength: T2 NE4, T2 NE3, and T2 NE5 ranked highest, whereas T1 PE ranked lowest. These findings identify persistent worry, hypervigilance, and stress-related symptoms as potential cross-community nodes linking early emotional abuse, alcohol use, and GI.

**Figure 5:**
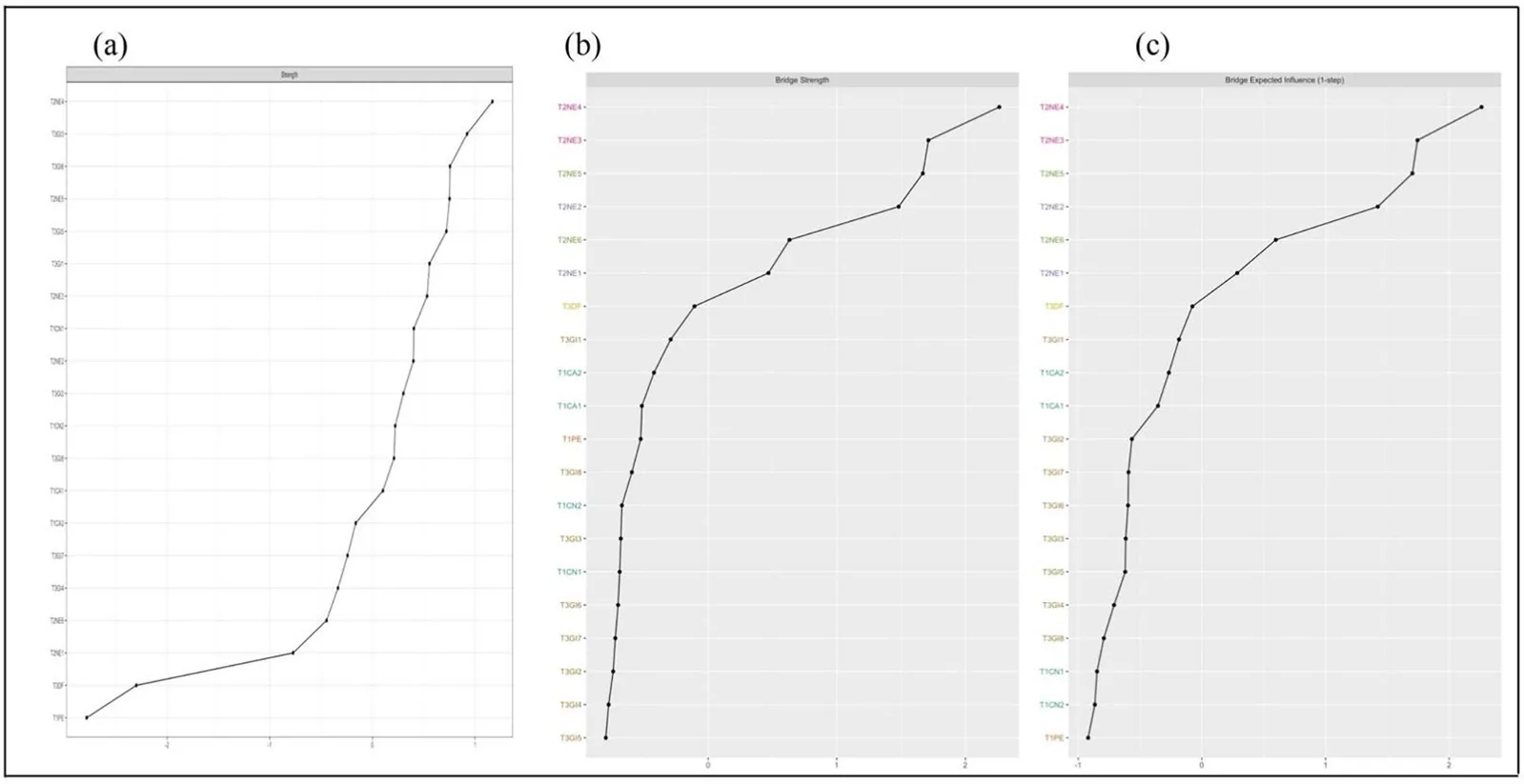
Node and bridge centrality estimates. The x-axis shows standardized z scores. **(a)** Node strength, which quantifies the overall connectivity of each node. **(b)** Bridge strength, which quantifies connections between a node and nodes in other variable communities. **(c)** One-step bridge expected influence, which additionally retains the direction of cross-community connections. CA, childhood emotional abuse; NE, negative emotion; DF, drinking frequency; PE, physical exercise; GI, gastrointestinal symptoms.

### 3.7 Stability of node strength

Node-strength estimates showed good stability, with a correlation-stability coefficient of CS (cor = 0.70) = 0.75; the corresponding case-dropping bootstrap results are provided in **Figure 6**.

**Figure 6:**
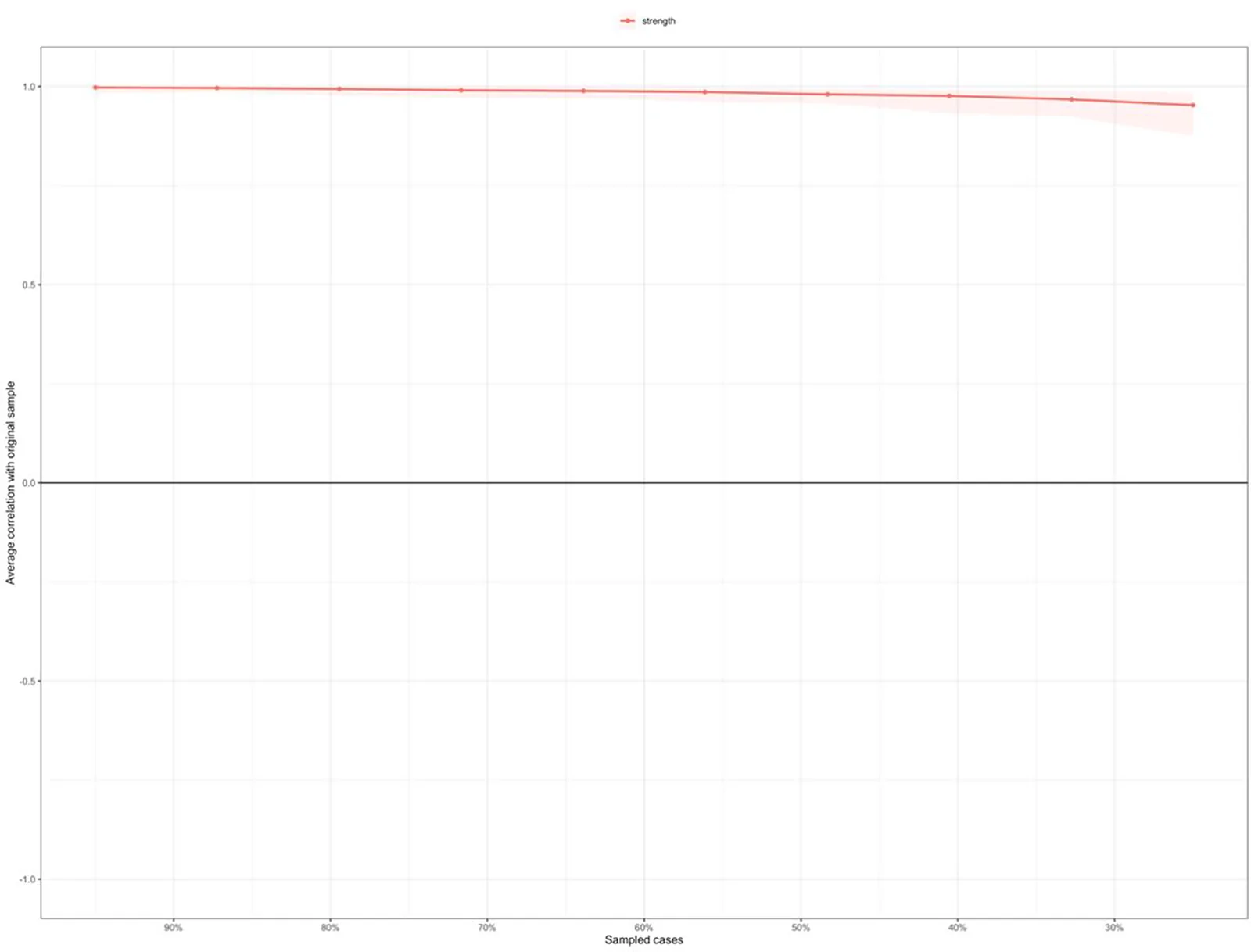
Case-dropping bootstrap analysis of node-strength stability. Note. The x-axis indicates the proportion of observations retained in each resampled subset, and the y-axis indicates the mean correlation between node-strength estimates from the subset network and the original network. Points and solid lines represent mean correlations; shaded areas represent 95% confidence intervals. The correlation-stability coefficient was CS (cor = 0.70) = 0.75. Values closer to 1 indicate greater stability in the ordering of node strength.

## 4 Discussion

This three-wave study yielded four main findings. First, CA was positively associated with subsequent GI after adjustment for demographic covariates. Second, NE represented the largest specific indirect association between CA and GI, whereas the indirect association through DF and the partially time-ordered serial association were considerably smaller. Third, higher PE attenuated, but did not eliminate, the positive association between CA and subsequent NE. Finally, the network analysis localized these variable-level associations to specific symptoms, with anxiety- and stress-related nodes showing prominent cross-community connectivity and DF showing a notable conditional association with upper abdominal pain or heartburn. Together, these findings suggest that emotional distress may be a particularly relevant correlate of gastrointestinal burden following childhood emotional adversity, while exercise and alcohol-related behaviors may represent potentially modifiable behavioral factors.

### 4.1 CA and GI

CA was positively associated with subsequent GI, and the association remained significant after adjustment for gender, age, and economic status, supporting H1 (83, 84). This result is consistent with evidence linking adverse childhood experiences to functional GI and indicates that the consequences associated with an adverse emotional environment in childhood may extend into physical health in early adulthood (85, 86). Unlike physical abuse, emotional abuse may leave no visible injury, yet persistent denigration, humiliation, rejection, threats, and inadequate emotional support can heighten threat sensitivity and sustain hypervigilance (62). Such prolonged stress responses may disturb hypothalamic-pituitary-adrenal activity, autonomic and immune regulation, and gut-brain communication, potentially affecting gastrointestinal motility, mucosal function, and visceral sensitivity (87, 88). Heightened attention to bodily sensations may further amplify otherwise mild gastrointestinal discomfort, particularly when those sensations are interpreted as threatening (89). During the transition to college, academic, interpersonal, and environmental demands may reactivate negative self-beliefs and stress-response patterns established earlier in life, allowing the association between CA and gastrointestinal discomfort to persist (32). The network analysis complemented this variable-level result by showing that the associations were not distributed uniformly across individual symptoms (90). The GI network also showed internal heterogeneity. GI2–GI4, representing reflux/nausea, rumbling/bloating, and belching/flatulence, were relatively strongly interconnected, whereas GI5–GI8 formed a second group centered on bowel habit and defecation-related complaints. This pattern suggests a relative separation between upper gastrointestinal or dyspeptic-type symptoms and defecation-related symptoms. Such organization may be clinically relevant because similar total GI scores could reflect different symptom profiles. However, these groupings represent conditional association patterns rather than clinically validated symptom clusters or diagnostic categories. The rejection-and-threat component of CA showed relatively prominent cross-community connectivity, consistent with the possibility that more explicit experiences of rejection or threat are closely related to persistent insecurity and threat anticipation (62). Anxiety- and stress-related nodes also showed notable links across childhood emotional abuse, drinking frequency, and GI (91). A comparatively strong conditional association between DF and upper abdominal pain or heartburn was also observed, although this edge neither establishes drinking as a coping response nor indicates its causal direction (91). Overall, the observed CA–GI association appears more consistent with an interplay of early adversity, emotional responses, and health-related behaviors than with a single explanatory process (92). These findings suggest that assessment of gastrointestinal complaints in college students may benefit from considering early emotional experiences alongside persistent psychological distress and relevant health behaviors.

### 4.2 Statistical indirect association through NE

NE showed a significant statistical indirect association between CA and GI, accounting for 36.23% of the total effect and supporting H2 (93). Notably, this was substantially larger than the indirect associations involving DF, indicating that NE may be a particularly important psychological correlate within the proposed model. One plausible explanation lies in difficulties in emotion regulation following persistent emotional maltreatment. Experiences of denigration, rejection, threat, or inadequate support may interfere with adaptive emotion regulation while fostering suppression, avoidance, rumination, negative self-appraisal, and heightened sensitivity to threat (29, 93, 94). When confronted with academic, interpersonal, or everyday demands, students with a history of CA may consequently be more likely to appraise events negatively and remain focused on bodily discomfort (89). Persistent worry and hypervigilance may increase attention to gastrointestinal sensations, while depressive symptoms may reduce motivation for adaptive health management and constructive coping (95). NE may thus statistically link early relational trauma with GI in early adulthood through a combination of negative appraisal, symptom-focused attention, and maladaptive coping (23). The network results further indicated heterogeneity in the connectivity of individual NE. Anxiety-related nodes occupied highly connected positions: NE4 had the highest node strength and bridge strength, while NE3 and the stress-related node NE5 also showed pronounced cross-community connectivity (96). Compared with an undifferentiated total score for NE, persistent worry, hypervigilance, and tension may therefore represent particularly relevant emotional components of the observed association between CA and GI (97). Taken together, the indirect-effect model and the symptom network were compatible with a sequence involving early emotional trauma, persistent NE, increased symptom-focused attention and dysregulated coping, and GI; they did not, however, establish this sequence as causal (90). Interventions addressing gastrointestinal health in college students may consequently benefit from assessing anxiety, tension, and persistent stress in addition to somatic complaints and from incorporating trauma-informed emotion-regulation support. From a network perspective, the relatively high node and bridge centrality of anxiety- and stress-related symptoms suggests that these symptoms may be useful candidates for prioritized screening and further intervention research because they were conditionally connected with multiple domains in the present network. However, centrality should not be interpreted as direct evidence that intervening on a highly central node will necessarily produce downstream improvement in other symptoms. Rather, these nodes identify clinically plausible points for further prospective and experimental evaluation.

### 4.3 Drinking frequency and the partially time-ordered serial association

DF showed a significant statistical indirect association between CA and GI, accounting for 5.10% of the total effect and supporting H3 (39). Compared with the indirect association through NE, however, its magnitude was considerably smaller. This pattern suggests that drinking frequency may represent a more limited and context-dependent behavioral correlate within the model. The results were consistent with a behavioral pathway in which CA was associated with more frequent alcohol use in early adulthood, which was in turn associated with GI (58). Because DF and GI were both measured at T3, this result does not establish that drinking preceded gastrointestinal symptoms or causally mediated the CA–GI association. Previous prospective research has likewise linked emotional abuse and emotional neglect with a greater likelihood of problematic drinking in young adulthood, supporting a temporal association between early relational adversity and later alcohol use (98). The present study differs from much of this literature because it focused on drinking frequency in a general college sample rather than alcohol use disorder or binge-drinking populations. Accordingly, the result should be interpreted specifically in terms of drinking frequency and not as evidence concerning problematic alcohol use more broadly. The relatively small indirect association is also consistent with evidence that drinking following childhood maltreatment varies according to individual and contextual factors (35, 37), including gender, peer environments, drinking motives, and individual susceptibility (99). The use of a single drinking-frequency item further narrows this interpretation. Although it efficiently captures how often drinking occurs, it cannot distinguish drinking quantity, binge-drinking episodes, beverage strength, or drinking context. Thus, higher-risk patterns may not be adequately represented by the present measure. Daily-monitoring studies indicate that binge drinking is more closely associated with subsequent diarrhea, nausea, stomach pain, and dyspepsia than lighter drinking, highlighting the importance of drinking dose and pattern (100, 101). Susceptibility (102), which may partly explain why DF accounted for only a relatively small proportion of the overall association. At the symptom level, DF showed relatively low overall centrality but a concentrated cross-community conditional association with T3 GI1, representing upper abdominal pain or heartburn (103). This result suggests that drinking frequency may be more closely associated with particular upper gastrointestinal complaints than with the GI domain as a whole. Importantly, the network edge represents conditional dependence after adjustment for other nodes and does not establish causal direction. Clinically, this localized association suggests that students who report both recurrent drinking and upper abdominal pain or heartburn may warrant more detailed assessment of alcohol quantity, drinking pattern, and gastrointestinal symptoms. This interpretation is intended to guide more focused assessment rather than to imply that reducing drinking frequency alone would necessarily alter gastrointestinal symptoms.

### 4.4 Serial indirect association through NE and drinking frequency

T2 NE and T3 DF formed a significant model-based serial indirect association between T1 CA and T3 GI, accounting for 2.03% of the total effect and supporting H4 (30). T1 CA was associated with higher levels of anxiety, depression, and stress at T2; T2 NE were subsequently associated with greater DF at T3; and T3 DF was associated with GI measured at the same wave (104). This was the smallest indirect effect observed in the model and, importantly, was only partially time-ordered because DF and GI were both assessed at T3. It should therefore be interpreted as a theoretically specified statistical association rather than evidence that drinking causally transmitted the effect of CA to GI. Despite its modest magnitude, the result is compatible with a developmental cascade perspective in which difficulties associated with early emotional adversity may extend from emotional adjustment to health-related behavior and, ultimately, physical health (45, 47, 51). Within this proposed sequence, CA may be associated with persistent NE, which in turn is associated with more frequent drinking and gastrointestinal discomfort (105). However, each link is likely to vary across individuals and situations (58). NE may be followed by rumination, avoidance, altered eating, active help-seeking, or other coping responses, while college drinking may occur for social rather than emotion-regulation reasons (106). Gastrointestinal responses may likewise vary according to diet, sleep, daily routines, and individual susceptibility (107, 108). Accordingly, only a subset of students may experience the full proposed sequence, making attenuation across successive associations plausible (47).

The network results offer a complementary symptom-level perspective without resolving this temporal limitation. Anxiety- and stress-related symptoms occupied notable cross-community positions (109), whereas DF showed a more localized conditional association with upper abdominal pain or heartburn (41). Thus, the serial model and network should not be treated as mutual confirmation: the former estimates an aggregate, partially time-ordered statistical association, whereas the latter identifies local conditional relationships among measured indicators. Neither establishes causal direction. Future daily-diary or ecological momentary assessment studies that repeatedly assess emotional states, drinking motives and patterns, and gastrointestinal responses would be better suited to testing the short-term ordering proposed here.

### 4.5 Moderating role of PE

T1 PE significantly moderated the association between T1 CA and T2 NE, supporting H5 (110). Simple-slope analyses showed that the positive association between T1 CA and T2 NE became progressively weaker as T1 PE increased (111, 112). PE therefore appeared to attenuate, rather than eliminate, the association between childhood emotional adversity and subsequent negative emotional symptoms. This pattern is consistent with the stress-buffering model (67). One possible explanation is that regular exercise provides a relatively controllable source of positive experience for individuals whose early environments were characterized by rejection, low support, or diminished perceived control (113). Exercise-related goal attainment may strengthen self-efficacy and bodily mastery while temporarily interrupting sustained attention to trauma-related experiences and current stressors (66, 114). Group exercise may additionally provide opportunities for positive interaction and social feedback (115). These processes may help explain why students reporting more frequent PE showed a weaker CA–NE association, although the present observational design does not establish the underlying mechanism. The moderation finding should also be interpreted within the limits of the PE measure. PE was assessed with a single frequency-based item; consequently, the present results do not identify whether particular exercise intensities, durations, or activity types provide stronger buffering effects. The network findings should be considered alongside, rather than as an explanation for, this moderation result. PE had relatively low node strength and bridge indices and did not emerge as a central connector across symptom communities (116). This is not inconsistent with its moderating role, because network centrality describes direct conditional connectivity within the observed symptom structure, whereas moderation assesses whether the association between two variables differs across levels of a third variable (117). The association between CA and NE remained significant even at high levels of PE. PE should therefore be understood as a potential supplementary buffering resource rather than a primary or stand-alone intervention capable of offsetting the consequences associated with early emotional trauma (118). Future studies using multidimensional or device-based assessments of exercise are needed to determine whether the observed buffering association varies by exercise frequency, intensity, duration, or type. In practice, regular PE may be most appropriately incorporated into a broader trauma-informed, multicomponent support framework that combines psychological support, emotion-regulation training, and alcohol-risk screening or management, rather than being used as an isolated intervention.

## 5 Limitations

Several limitations should be considered. First, convenience sampling was used, and participants were recruited mainly from universities in four regions of central and western China. Although the sample included multiple institutions and academic years, it may not represent students in other regions or cultural settings, and caution is warranted when generalizing the findings to nonstudent populations. Cultural and educational contexts may also shape how childhood emotional experiences and psychological distress are perceived, recalled, and reported, as well as opportunities for drinking, exercise, help-seeking, and access to health services. Institutional differences in campus drinking norms, physical-activity opportunities, psychological support, and health resources may therefore contribute to variation in the prevalence or expression of the constructs examined here. These contextual factors were not directly measured in the present study, so their contribution cannot be determined. Participants were also nested within universities and may have shared aspects of the campus environment, health resources, and drinking culture. Because this clustering was not explicitly modeled, the assumption of independent observations may have been imperfect, potentially leading to underestimated standard errors and overly precise confidence intervals. Future studies should recruit participants from more diverse cultural and educational settings and use multilevel models with school-level contextual measures to account explicitly for institutional clustering. Second, childhood emotional abuse, NE, PE, drinking frequency, and GI were all assessed by self-report and may therefore be affected by recall bias, social-desirability bias, and common method variance. Reports of childhood experiences were retrospective, and the influence of current emotional states on memory retrieval cannot be excluded. GI were not evaluated against clinical diagnoses, so functional complaints could not be distinguished from symptoms associated with organic disease. Accordingly, the GI score should be interpreted as self-reported gastrointestinal symptom burden and should not be used to infer the presence or prevalence of IBS or other functional gastrointestinal disorders in this sample. No physiological indicators of HPA-axis function, autonomic activity, inflammatory responses, intestinal barrier function, or gut microbiota were collected. These proposed processes therefore could not be tested in the present study and require confirmation in future research incorporating relevant biomarkers and other objective measures. Third, although the three-wave design provided temporal separation for some variables, the principal constructs were not repeatedly measured at every wave. Baseline levels could therefore not be adequately controlled, and reverse associations—for example, GI influencing emotional states or health behaviors—remain possible. Most importantly, DF and GI were both measured at T3. Their temporal ordering cannot be determined, and the serial mediation findings represent model-based statistical indirect associations arranged according to theory rather than evidence that DF preceded GI. The results are better interpreted as partially time-ordered longitudinal associations than as causal effects. Fourth, DF and PE were each measured with a single item. Although these brief indicators reduced response burden and were practical for a large, multi-wave survey, they did not permit estimation of internal consistency and provided less comprehensive coverage than multidimensional measures. The alcohol item did not distinguish the amount consumed per occasion, binge drinking, beverage type, or drinking motives, while the PE item did not fully characterize intensity, duration, or activity type. These measurement constraints limit the precision with which behavioral processes can be interpreted. Future work should use multidimensional instruments, daily diaries, or ecological momentary assessment to capture short-term changes in NE, drinking, and GI. Finally, the network analysis estimated conditional associations after accounting for all other nodes. Neither node centrality nor bridge indices establish causal direction. Although the stability analysis supported the robustness of node-strength rankings, the network structure requires replication in independent samples, and longitudinal network or experimental studies are needed to determine whether the identified nodes play a prospective role in symptom development.

## 6 Conclusion

At both the variable and symptom-network levels, this study identified associations linking CA with GI among college students. CA was directly associated with subsequent GI and showed separate statistical indirect associations through NE and drinking frequency, as well as a small serial indirect association involving NE, drinking frequency, and GI. Because DF and GI were assessed concurrently at T3, this serial association was only partially time-ordered and should not be interpreted as a causal pathway. The positive association between CA and NE was weaker at higher levels of PE, consistent with a potential buffering role. The network results further showed that anxiety- and tension-related nodes occupied prominent connecting positions across constructs and that DF had a notable conditional association with upper abdominal pain or heartburn; these edges describe conditional dependence rather than causal influence. Taken together, the findings support further evaluation of trauma-informed, multicomponent approaches in college health services that combine recognition of early adversity with emotion management, alcohol-risk screening, and regular PE to address gastrointestinal and related psychological burdens among students.

## Data Availability

The raw data supporting the conclusions of this article will be made available by the authors, without undue reservation.
